# Gut-Evolved *Candida albicans* Induces Metabolic Changes in Neutrophils

**DOI:** 10.3389/fcimb.2021.743735

**Published:** 2021-11-22

**Authors:** Jose Antonio Reales-Calderon, Gloria H. W. Tso, Alrina S. M. Tan, Pei Xiang Hor, Julia Böhme, Karen W. W. Teng, Evan W. Newell, Amit Singhal, Norman Pavelka

**Affiliations:** ^1^ Singapore Immunology Network (SIgN), Agency for Science, Technology and Research (A*STAR), Singapore, Singapore; ^2^ A*STAR Infectious Diseases Labs (ID Labs), Agency for Science, Technology and Research (A*STAR), Singapore, Singapore

**Keywords:** *Candida albicans*, neutrophils, trained immunity, infectious diseases, live attenuated vaccines, immunometabolism

## Abstract

Serial passaging of the human fungal pathogen *Candida albicans* in the gastrointestinal tract of antibiotics-treated mice selects for virulence-attenuated strains. These gut-evolved strains protect the host from infection by a wide range of pathogens *via* trained immunity. Here, we further investigated the molecular and cellular mechanisms underlying this innate immune memory. Both Dectin-1 (the main receptor for β-glucan; a well-described immune training molecule in the fungal cell wall) and Nod2 (a receptor described to mediate BCG-induced trained immunity), were redundant for the protection induced by gut-evolved *C. albicans* against a virulent *C. albicans* strain, suggesting that gut-evolved *C. albicans* strains induce trained immunity *via* other pathways. Cytometry by time of flight (CyTOF) analysis of mouse splenocytes revealed that immunization with gut-evolved *C. albicans* resulted in an expansion of neutrophils and a reduction in natural killer (NK) cells, but no significant numeric changes in monocytes, macrophages or dendritic cell populations. Systemic depletion of phagocytes or neutrophils, but not of macrophages or NK cells, reduced protection mediated by gut-evolved *C. albicans*. Splenocytes and bone marrow cells of mice immunized with gut-evolved *C. albicans* demonstrated metabolic changes. In particular, splenic neutrophils displayed significantly elevated glycolytic and respiratory activity in comparison to those from mock-immunized mice. Although further investigation is required for fully deciphering the trained immunity mechanism induced by gut-evolved *C. albicans* strains, this data is consistent with the existence of several mechanisms of trained immunity, triggered by different training stimuli and involving different immune molecules and cell types.

## Introduction

Immunological memory is classically ascribed to T and B cells, but protection against reinfection has been observed also in organisms that lack adaptive immunity, such us invertebrates and plants (Durrant and Dong, 2004) as well as Recombination activating gene 1 (Rag1) knock-out (KO) mice (Quintin et al., 2012). This phenomenon is referred to as ‘trained immunity’, defined as an enhanced state of the innate immune system after primary exposure of a host to certain pathogens or molecules, which results in stronger secondary innate responses and greater protection of the host against reinfections (Netea et al., 2011). For instance, infection of mice by murine cytomegalovirus (MCMV) expands a specific subset of NK cells, characterized by memory-like properties and by an epigenetically poised state, that confers protection against further infections by the same virus (Sun et al., 2009; Lau et al., 2018). Moreover, the Bacille Calmette-Guerin (BCG) strain of *Mycobacterium bovis* induces trained immunity by a mechanism that is dependent on i) NK cells, (ii) epigenetically reprogrammed monocytes and (iii) the nucleotide-binding oligomerization domain-containing protein 2 (Nod2) receptor (Kleinnijenhuis et al., 2012; Kleinnijenhuis et al., 2014). In addition, BCG vaccination also leads to long-term reprogramming of human neutrophils, as shown recently (Moorlag et al., 2020). Similarly, immunization with sublethal doses of the human fungal pathogen *Candida albicans* or with the fungal cell wall component β-glucan protects mice from secondary infection with a fully virulent strain of *C. albicans* (Quintin et al., 2012), *via* a mechanism involving epigenetic and metabolic reprogramming of monocytes and macrophages (Cheng et al., 2014; Saeed et al., 2014).

In a previous study, we described that adaptive evolution of a fully virulent strain of *C. albicans* in the gastrointestinal tract of antibiotic-treated mice leads to reproducible selection of genetic variants with two key characteristics. First, all gut-evolved *C. albicans* strains lost the yeast-to-hyphae morphogenetic program and its associated virulence. Secondly, these evolved fungal strains gained the ability to train the host innate immune system with high efficiency (Tso et al., 2018). In particular, immunization of mice with gut-evolved *C. albicans* protected the host from subsequent challenge with fungi (fully virulent *C. albicans* or the filamentous fungus *Aspergillus fumigatus*) or bacteria (the Gram-positive *Staphylococcus aureus* or the Gram-negative *Pseudomonas aeruginosa*). Consistent with trained immunity, we observed this protection also in Rag1 KO mice, clearly implicating an innate memory mechanism.

In addition to epigenetic changes, trained immunity also rewires metabolic pathways, which leads to changes in cell physiology and a long-term pro-inflammatory phenotype (Netea et al., 2016). In β-glucan-trained monocytes, a shift from oxidative phosphorylation towards aerobic glycolysis occurs, leading to high glucose consumption and increased lactate production (Cheng et al., 2014). Importantly, Dectin-1, the main receptor of β-glucan (Brown et al., 2002), is essential for β-glucan to induce this metabolic shift. In contrast, BCG-trained monocytes demonstrate an increase in not only the basal and maximal extracellular acidification rate (ECAR), a measure of lactate production, but also in the oxygen consumption rate (OCR), a measure of oxidative phosphorylation (Arts et al., 2016), indicating that different training stimuli might induce different metabolic changes in innate immune cells.

In this study, we characterized further the cellular and molecular mechanisms underlying the trained immunity induced by gut-evolved *C. albicans*. First, we tested the involvement of various pathogen-recognition receptors but none, including Dectin-1 and Nod-2, was completely required for training and protection against secondary infections. We next employed a comprehensive mass cytometry (CyTOF) panel and characterized myeloid cells from spleen of mice immunized with gut-evolved *C. albicans* strains. We observed a significant increase of neutrophil numbers at the expense of NK cells. Depletion of various innate immune cell populations *in vivo*, in gut-evolved *C. albicans* immunized mice, identified an important role of phagocytes in general and of neutrophils in particular, but not of macrophages or NK cells, in mediating protection against secondary challenges with virulent *C. albicans* or *P. aeruginosa*. Finally, we employed Seahorse technology to investigate the bioenergetic changes induced during *in vivo* training with gut-evolved *C. albicans*, and reported an increased glycolysis as well as respiration in the spleen and in bone marrow cells, and in particular in splenic neutrophils. Together, our data show that gut-evolved *C. albicans* strains induce a trained immunity program that shares similarities and differences with previously described ones.

## Materials and Methods

### Microbial Strains and Growth Conditions

We grew all *C. albicans* strains (SC5314, W2N, R24) in yeast extract-peptone-dextrose (YPD) media supplemented with 2% glucose, and *P. aeruginosa* (ATCC 14206) in Luria-Bertani (LB) broth. All cultures were shaken at 200 rpm and incubated at 37°C overnight.

### Mice

Six-to-eight-weeks-old male or female C57BL/6J (wild type), Dectin1^−/−^, NOD2^−/−^, CCR2^−/−^ (Jackson Laboratories), TLR^−/−^ or TLR4^−/−^ (OBS Japan) mice reared under specific pathogen free conditions were used in all experiments. Mice were randomly assigned to the different groups in all the experiments.

### Protection and Cross-Protection Assays

Six-to-eight-weeks-old wild-type (WT) or Dectin1^−/−^, NOD2^−/−^, TLR2^−/−^, TLR4^−/−^ and CCR2^−/−^ mice were immunized *via* tail-vein injection of 5×10^5^ colony-forming units (CFUs) of live *C. albicans* R24 strain or with phosphate buffered saline (PBS) as a mock-immunization control. We then performed a secondary systemic challenge with a lethal dose (5×10^5^ CFUs) of the WT, fully virulent strain SC5314 at day 14 after the primary immunization. For cross-protection experiments, mice were challenged with a lethal dose of *P. aeruginosa* (2.5×10^7^ CFUs) at day 14 post-immunization. In all experiments, we monitored and recorded mouse survival daily. Mice were euthanized by CO_2_ followed by cervical dislocation when they reached moribund condition.

### Cytometry by Time-Of-Flight

We immunized mice *via* tail-vein injection with 5×10^5^ CFUs of the evolved strains R24 or W2N, with a sublethal dose of the WT *C. albicans* SC5314 (1×10^4^ CFUs) or with phosphate buffered saline (PBS) as a mock-immunization control. After 28 days, we isolated splenocytes from the immunized mice, performed red blood cells lysis and depleted CD19 and CD90.2 with microbeads (Miltenyi), as well as removed dead cells by dead cells removal kit (Miltenyi).

Sample preparation. We stained the isolated cells with 100 µL of 50 µM of cisplatin (Sigma-Aldrich, cat. no. 479306-1G) at 4°C. After 5 minutes, we washed cells with staining buffer (1× PBS + 4% fetal bovine serum [FBS] + 0.05% sodium azide) and then incubated with anti-MerTK-Biotin and anti-CCR2-APC for 30 min at 4°C. We washed cells twice with staining buffer, followed by 30 min incubation in metal isotope-tagged surface antibodies at 4°C (see **Table S1** for the complete list of antibodies). We then washed cells twice with staining buffer, once with PBS, and then fixed in 2% paraformaldehyde (Electron Microscopy Sciences, cat. no. 15710) in PBS at 4°C overnight. The next day, cells were pelleted and incubated in 1× permeabilization (perm) buffer for 5 min on ice. We then washed cells once with PBS and incubated them with cellular barcodes on ice as previously described (Wong et al., 2015). After 30 min, we washed cells once with perm buffer, re-suspended them in staining buffer and incubated them for 10 min on ice. We then labeled cellular DNA at room temperature with 250 nM iridium intercalator (Fluidigm Corporation, cat. no. 201192B) in 2% PFA/PBS for 20 min. Finally, we washed cells twice with staining buffer and ultrapure (Milli-Q) water prior to sample acquisition.

Data acquisition. First, we pooled cells from all samples, and then enumerated, filtered and diluted them to a final concentration of 6 × 10^5^ cells/ml. Mass-tag barcoding was used in this experiment to acquire all samples simultaneously. Before acquisition, we added EQ Four Element Calibration Beads (Fluidigm Corporation, cat. no. 201078) to the pooled samples at a final concentration of 1%. Samples were acquired on a CyTOF2 (Fluidigm Corporation) which was equipped with a Super Sampler fluidic system (Victorian Airship & Scientific Apparatus LLC) at an event rate of <500 events per second.

Data analysis. After sample acquisition, .FCS files were exported and normalized as previously described (Finck et al., 2013). We then assigned a random value from a uniform distribution between −1 and 0 to all events associated with parameters having zero values. Each sample containing a unique combination of 2 metal barcodes was deconvoluted by Boolean gating using Flowjo Software (Tree Star, Ashland, USA), followed by manual gating to exclude residual beads, debris and dead cells. After data pre-processing, we reduced the dimensionality of the data using the t-Distributed Stochastic Neighbor Embedding (t-SNE) algorithm (Guilliams et al., 2016) using a custom R script based on the “flowcore” and “Rtsne” CRAN R packages as previously described (Becher et al., 2014;Wong et al., 2015). All data was transformed using the “logicalTransform” function and w=0.25, t=16409, m=4.5 and a=0 as input parameters. Finally, we performed manual gating on the t-SNE clusters and assigned cluster numbers arbitrarily.

### *In Vivo* Depletion of Immune Cell Populations

WT mice were immunized *via* intravenous injection of 5×10^5^ CFUs of live R24 *C. albicans*. Prior to the secondary challenge with a lethal dose (5×10^5^ CFUs) of WT virulent SC5314 at day 14, we performed one of the following cell depletions. To deplete the phagocytes, clodronate liposome suspension (5 mg/ml) was diluted 1:5 in PBS and administered 200 μl intravenously every other day, starting 7 days prior to infection. To deplete macrophages, we injected mice with 0.2 mg of anti-CSF1R neutralizing monoclonal antibody (BioXcell) or an isotype-matched control antibody (anti-rat IgG2a) intravenously 4 days before challenge and administered every other day until the end of the experiment. To deplete NK cells, we injected 0.2 mg of anti-NK1.1 neutralizing monoclonal antibody (BioXcell) or an isotype-matched control antibody (anti-rat IgG2a) intravenously 4 days prior the challenge and every other day until the end of the experiment. To deplete neutrophils, we injected mice with 0.2 mg of anti-Ly6G neutralizing antibody (BioXcell) or an isotype-matched control antibody (anti-rat IgG2a) 4 days prior the challenge, on the day of challenge and 4 days after the challenge.

### Analysis of Cellular Metabolic Function

Seahorse XFe96 Cell Mito Stress and Glycolytic Rate Tests (Seahorse, Agilent Technologies, Santa Clara, CA) were performed according to the manufacturer’s instruction on a SeaHorse XF Extracellular Flux Analyzer (Agilent). Spleens and bone marrows of mice immunized *via* tail-vein injection of 5x10^5^ CFUs of live *C. albicans* R24 strain were harvested and homogenized 14 days after the immunization. The cell suspension was centrifuged and resuspended in red cell lysis buffer (BioLegend) for 5 min. We washed cells twice in PBS, and then solubilized splenocytes or bone marrow cells in either Mitostress complete media or in Glycolytic rate media. Mitostress complete media consisted of XF Assay Medium (Seahorse Bioscience), supplemented with 25 mM glucose, 2 mM L-glutamine, 2 mM sodium pyruvate, 2% heat-inactivated FBS and 1% Penicillin-Streptomycin (Pen/Strep). Glycolytic rate media consisted of XF Assay Medium (Seahorse Bioscience), supplemented with 10 mM glucose, 2 mM L-glutamine, 1 mM sodium pyruvate, 5mM Hepes and 1% Pen/Strep. We then seeded cells on a sterile XFp plate in quintuplicate at 6×10^5^ total cells per well, and incubated them for 1 h at 37°C without CO_2_. After the incubation, cells undergoing the Mitostress assay were serially stimulated in the following sequence: (A) 1 μM Oligomycin; (B) 1 μM carbonyl cyanide-4-(trifluoromethoxy) phenylhydrazone (FCCP); (C) 0.5 μM Rotenone/Antimycin A. For the glycolytic rate assay, cells were stimulated with (A) 0.5 μM Rotenone/Antimycin A (B) 50 mM 2-Deoxy-D-glucose (2DG).

In subsequent experiments, we first sorted neutrophils by FACS from spleens and bone marrows. Briefly, mice femurs, tibias and pelvic bones were crushed in PBS containing 2mM EDTA and 2% FBS and passed through a 70–μm nylon mesh sieve. Spleens were harvested and homogenized into single-cell suspensions using 70–μm nylon mesh sieves and syringe plungers. For the identification of bone marrow and spleen neutrophils, cells were stained with fluorophore-conjugated anti-mouse antibodies against CD11b (M1/70) and Ly-6G (1A8). Sorting of neutrophils was performed using a BD ARIAII (BD) to achieve >98% purity. After the sorting, mitochondrial stress analysis was performed on the sorted cells.

Respiratory parameters were obtained as the mean value ± standard error of the mean (SEM) for each time point in pmol per minute (n = 5 mice and 5 technical replicates per group) and included basal oxygen consumption rate (OCR, an indicator of oxidative phosphorylation), extracellular acidification rate (ECAR, an indicator of glycolysis), basal respiratory capacity, ATP production, maximal respiratory capacity, spare respiratory capacity, basal glycolysis and compensatory glycolysis. Data analysis was performed using the Seahorse Wave software.

### Statistical Analyses

All statistical analysis was performed in GraphPad Prism v9. All values are expressed as means ± SEM. *P* < 0.05 was considered as statistically significant.

## Results

**Figure 1:**
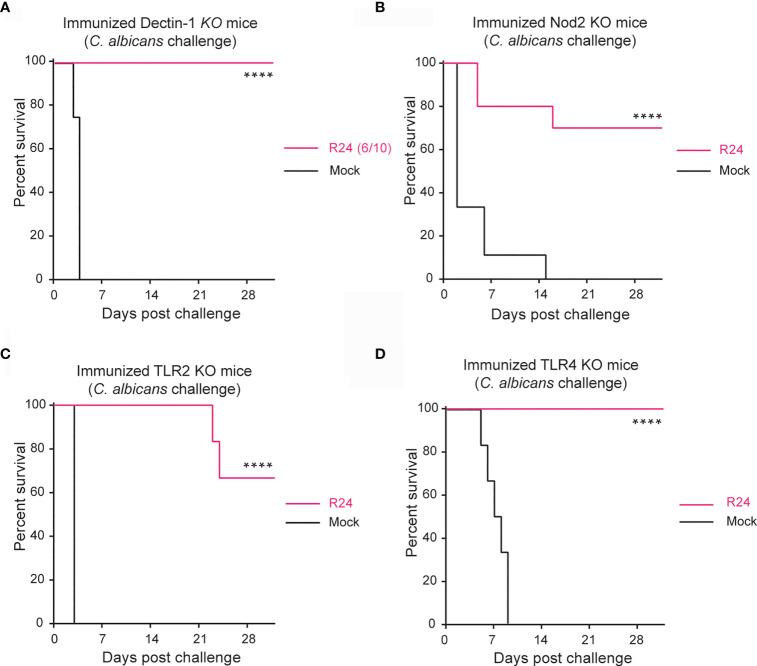
Protection against systemic candidiasis using the evolved strain is not dependent on Dectin-1, NOD2, TLR2 or TLR4. Survival of Dectin-1 **(A)**, NOD2 **(B)**, TLR2 **(C)** and TLR4 **(D)** KO mice immunized with 5x10^5^ CFUs of the gut-evolved strain R24 were challenged at day 14 post-immunization with a lethal dose of the WT *C. albicans* (SC5314) (5x10^5^ CFUs). n = 10 mice/group. Log-rank survival test was performed in Prism v9. ****P < 0.0001.

To dissect the molecular mechanism of trained immunity induced by gut-evolved *C. albicans*, we immunized mice bearing knock-out (KO) mutations in various receptors previously implicated in trained immunity, with a gut-evolved attenuated strain of *C. albicans* (termed strain R24). These mice were then challenged with a fully virulent strain of *C. albicans* (SC5314) at day 14 post-immunization. Consistent with Dectin-1 playing a crucial role in the innate immune defense against fungi (Taylor et al., 2007), some of the Dectin-1 KO mice (4 of 10) did not survive the primary immunization with R24 (**Figure S1**). Surprisingly, however, all six mice that survived the primary immunization with R24 also survived the secondary challenge with the lethal dose of SC5314 strain, indicating that Dectin-1 is not involved in the protective mechanism of trained immunity induced by gut-evolved *C. albicans* R24 (**Figure 1A**). We repeated a similar experiment in Nod2 KO mice and observed that 70% of the R24 immunized mice survived the lethal challenge with SC5314 *C. albicans*, indicating that gut-evolved *C. albicans* is still able to confer a significant protection to host animals even in the absence of this receptor (**Figure 1B**).

Toll-like receptor 2 (TLR2) is involved in antifungal immune responses (Netea et al., 2004; Tessarolli et al., 2010) and implicated with trained immunity against the spirochete *Leptospira interrogans* (Santecchia et al., 2019). We therefore next tested the role of TLR2, but again found that gut-evolved *C. albicans* strain R24 was able to confer a significant protection (70% *vs.* 0% survival) also to SC5314 challenged TLR2 KO mice (**Figure 1C**). As a control, we also repeated the experiment in TLR4 KO mice, bearing in mind that TLR4 is the receptor for lipopolysaccharide (LPS), which induces immune tolerance and acts antagonistically to β-glucan-induced immune training (Novakovic et al., 2016). As expected, R24-immunized TLR4 KO mice were completely protected from systemic infection by SC5314 virulent strain of *C. albicans*. Taken together, these results indicate that host protection induced by immunization with a gut-evolved *C. albicans* (**Figure 1D**) strain is independent of innate receptors so far associated with trained immunity.

**Figure 2:**
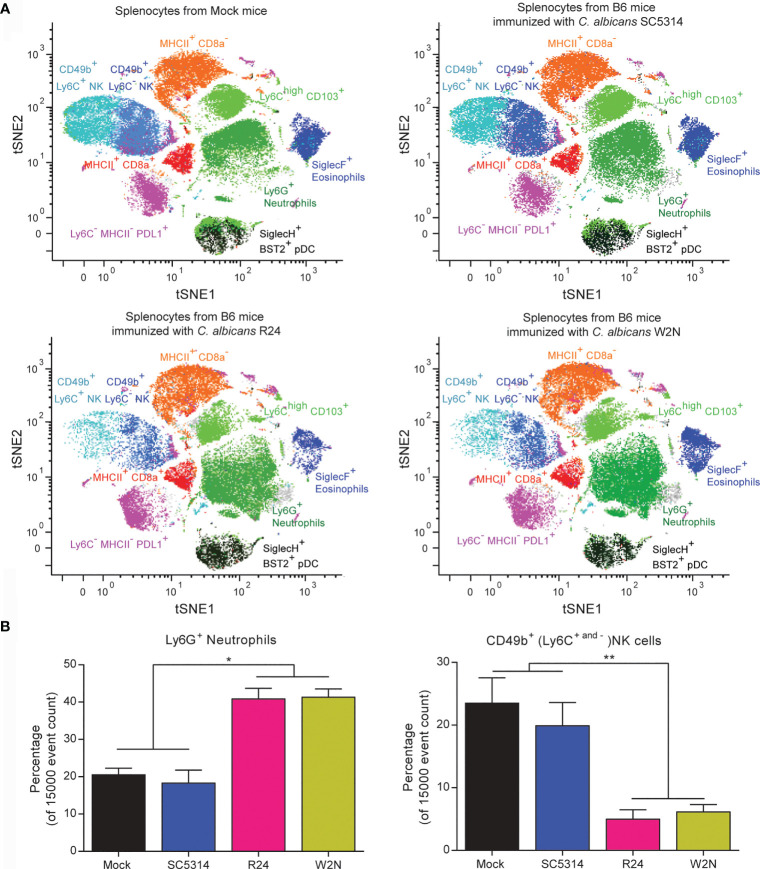
Cytometry by Time-Of-Flight (CyTOF) analysis of splenocytes from mice vaccinated with the evolved strains R24 and W2N identifies multiple changes in the innate immunity populations. **(A)** tSNE plots of CD45^+^CD90^−^CD3^−^CD19^−^ myeloid spleen cells from non-vaccinated mice and mice vaccinated with a low dose of the wild-type *C. albicans* strain SC5314 (10^4^ CFUs/mouse) or with the evolved *C. albicans* strains R24 and W2N (5x10^5^ CFUs/mouse) illustrating color-coded cell populations that clustered based on cell surface marker expression. **(B)** Bar graphs show the mean ± SEM of the Neutrophils and NK populations in the splenocytes from vaccinated and non-vaccinated mice. n=3 mice/group. One-way ANOVA with multiple comparisons (ANOVA p-value of 0.0002 for neutrophils and 0.0031 for NK cells), **P < 0.01, *P < 0.05.

In our previous work, we reported that immunization with the gut-evolved R24 and W2N *C. albicans* strains (but not with a sublethal dose of the WT *C. albicans* SC5314 strain) protected hosts against secondary lethal challenge with either WT SC5314 *C. albicans* or other fungal and bacterial species (Tso et al., 2018). This was true even in Rag1 KO mice that lack functional T and B cells. We also showed that this protection and cross-protection correlated with a significantly increased ability of splenocytes to produce IL-6 upon restimulation *ex vivo*. However, this was true only if we immunized the mice with gut-evolved *C. albicans* 28 days prior to splenectomy, and not if we immunized the mice with a sublethal dose of WT SC5314 *C. albicans* (Tso et al., 2018). This marked functional difference in splenocytes offered an opportunity to screen for cells and pathways underlying the trained immunity program induced by the R24 strain. Therefore, we used a panel of 38 antibodies (**Table S1**) and the CyTOF technology to characterize the myeloid cell compartment in splenocytes of mice 28 days post-immunization with either gut-evolved R24 or W2N, or with WT SC5314 *C. albicans*. Analysis of CD45^+^CD90^−^CD3^−^CD19^−^ myeloid spleen cells using nonlinear dimensionality reduction (tSNE) in conjunction with DensVM algorithm [a machine-learning clustering approach (Becher et al., 2014)], identified 9 distinct innate cell populations: Ly6G^+^ neutrophils, CD49b^+^Ly6C^+^ NK cells, CD49b^+^Ly6C^−^ NK cells, Ly6C^high^CD103^+^ monocytes, MHCII^+^CD8a^+^ dendritic cells (DCs), MHCII^+^CD8a^−^ DCs, SiglecH^+^BST2^+^ plasmacytoid DCs, SiglecF^+^ eosinophils, and a population of Ly6C^−^MHCII^−^PDL1^+^ cells (**Figure 2A**). We observed global differences in innate cell subpopulations in splenocytes from mice vaccinated with the gut-evolved strains R24 and W2N and the non-vaccinated mice or the mice vaccinated with the sublethal dose of WT *C. albicans*, including large shifts in the relative frequencies, as well as some subtle but reproducible shifts in high-dimensional phenotypes (see shifts on tSNE plots in **Figure 2A**) of several cell populations, predominantly Ly6G^+^ neutrophils, Ly6C^high^CD103^+^ monocytes and Ly6C^−^ MHCII^−^ PDL1^+^ cells (**Figure 2A**). One of the major differences that we observed was a significant increase in the relative frequency of Ly6G^+^ neutrophils and a concurrent significant decrease in the relative frequency of CD49b^+^ (Ly6C^+^ and Ly6C^−^) NK cells in mice immunized with a gut-evolved R24 and W2N strain compared to those immunized with the WT SC5314 strain and the non-vaccinated mice (**Figure 2B**). No significant numerical differences were found in other cell populations (**Figure S2**).

**Figure 3:**
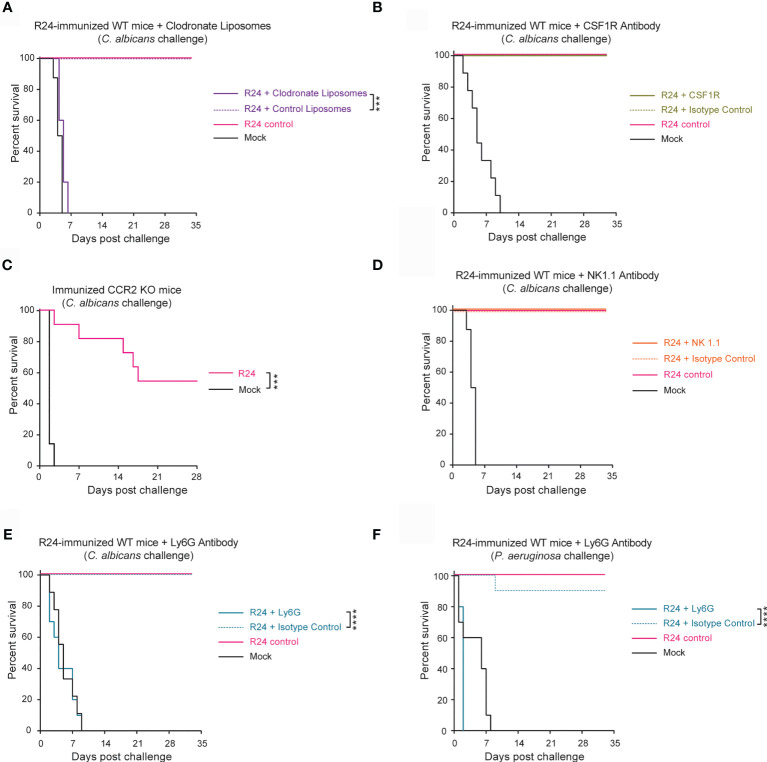
Gut-evolved *C. albicans* R24-induced protection from systemic fungal infections is mediated by neutrophils. **(A)** WT mice systemically immunized with *C. albicans* R24 were injected at day 7 post-immunization with either clodronate liposomes or control liposomes (purchased from http://clodronateliposomes.com) i.v. at 200 µl liposome suspension/mouse every other day. Animals were challenged at day 14 post-immunization with the lethal dose of the WT *C. albicans* (SC5314) (5x10^5^ CFUs). 100% of mice immunized with the gut evolved strain (R24) succumbed after systemic challenge by WT SC5314 when treated with clodronate liposomes, but not with the control liposomes. Macrophages were specifically depleted using CSF1R neutralizing antibody **(B)** and the role of monocyte recruitment was studied in CCR2^−/−^ mice **(C).** NK cells and neutrophils were specifically depleted using NK1.1 neutralizing antibody **(D)** or Ly6G neutralizing antibody **(E)**, respectively. None of the immunized mice succumbed after systemic challenge by WT SC5314 when treated with CSF1R or NK1.1 antibodies, while the immunized mice treated with the Ly6G neutralizing antibody succumbed to the challenge with a lethal dose of the WT *C. albicans* **(E)**, or with a lethal dose of *P. aeruginosa* **(F)**. n = 10 mice/group. Log-rank survival test was performed in Prism v9. ***P < 0.001, ****P < 0.0001.

To assess which innate immune cell populations were responsible for the protection induced by R24-mediated trained immunity, we depleted several major cell types in R24-immunized mice prior to challenge with a lethal dose of WT SC5314 *C. albicans* (**Figure 3**). To deplete overall phagocyte populations, we administered clodronate liposomes 7 days before lethal challenge (**Figure S3A**). All mice treated with clodronate liposomes succumbed to the challenge, while all mice treated with PBS-containing liposomes survived the infection (**Figure 3A**). Interestingly, the depletion of macrophages using CSF1R neutralizing antibody (**Figure S3B**) had no effect on the protection of the R24 immunized mice against challenge by SC5314 *C. albicans* (**Figure 3B**), whereas the vaccination of CCR2^−/−^ mice with the evolved strain R24 showed a partial protection against the systemic challenge with the virulent strain SC5314 (**Figure 3C**). Consistent with the reduced, as opposed to increased, number of NK cells in the spleens of R24 trained mice (**Figure 2B**), the depletion of NK cells using the NK1.1 neutralizing antibody (Figure S3C) similarly failed to affect the protection against systemic candidiasis (**Figure 3D**). Finally, the depletion of neutrophils using the Ly6G neutralizing antibody (**Figure S3D**) completely abrogated the protection of the R24-vaccinated mice when challenged with *C. albicans* SC5314 (**Figure 3E**) or with a lethal dose of *Pseudomonas aeruginosa* (**Figure 3F**). Altogether, these results indicate that the protective innate immune response acquired by vaccination with *C. albicans* R24 is dependent on the presence, at the time of the secondary challenge, of neutrophils in particular, but not of macrophages or NK cells. Furthermore, monocytes, instead, played an important but only partial role in this system.

**Figure 4:**
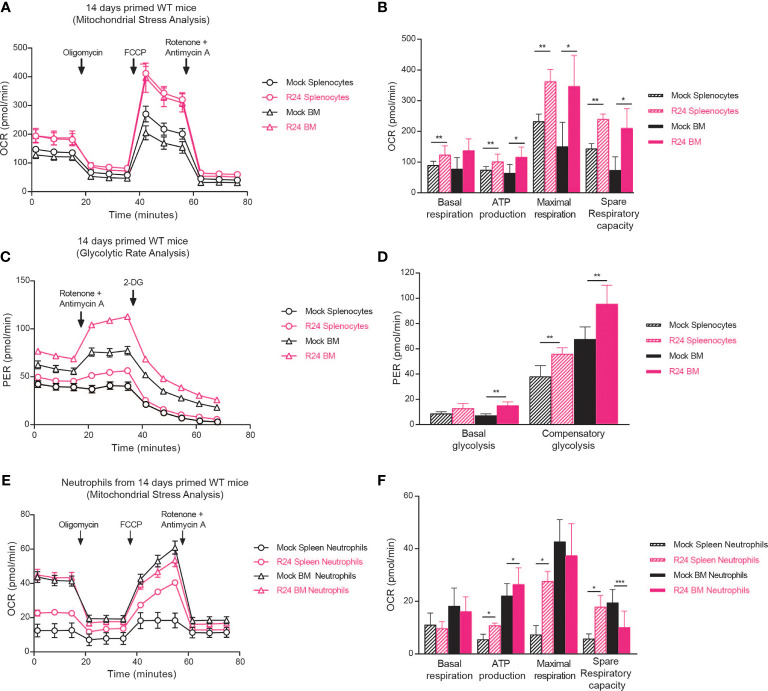
R24 vaccination increase both mitochondrial respiration and glycolytic rate/potential. **(A)** *Ex vivo* Oxygen consumption rate (OCR) in Splenocytes and Bone Marrow cells from mice vaccinated 14 days with the gut evolved strain R24 (5x10^5^ CFUs) and non-vaccinated mice. Splenocytes and BM cells were purified, plated in 96-well plates and exposed to oligomycin, FCCP, and ROT plus AA (see “Materials and methods”). OCR was measured as pmol O_2_/min/µg. **(B)** Proton Efflux Rate (PER) in Splenocytes and Bone Marrow cells from mice vaccinated 14 days with the gut evolved strain R24 (5x10^5^ CFUs) or non-vaccinated mice was determined by the glycolytic rate assay as mpH/min. **(C)** Basal Respiration, ATP production, maximal respiration and spare respiratory capacity were obtained by the mitochondrial stress analysis and are represented as mean ± SEM of n = 5 individual samples. **(D)** Basal glycolysis and compensatory glycolysis were obtained by the glycolytic rate assay and are represented as mean ± SEM of n = 5 individual samples. **(E)** *Ex vivo* Oxygen Consumption Rate (OCR) in neutrophils sorted from spleens from R24 vaccinated mice and non-vaccinated mice. **(F)** Basal Respiration, ATP production, maximal respiration and spare respiratory capacity are represented as mean ± SEM of n = 5 individual samples. Mann-Whitney tests between R24-vaccinated and mock-immunized mice were performed using Prism v9. *P < 0.05, **P < 0.01, ***P < 0.001.

Trained immunity is associated with and dependent on metabolic changes in innate immune cells (Saeed et al., 2014; Arts et al., 2016). Having observed neutrophil-dependent trained immunity in R24-vaccinated mice (**Figure 3E, F**), we asked if immunization with *C. albicans* R24 induced metabolic changes in neutrophils. Because neutrophils’ half-life is in the order of hours or days (Hidalgo et al., 2019), while trained immunity by gut-evolved *C. albicans* can last for up to 3 months (Tso et al., 2018), we first measured metabolic changes, in particular mitochondrial respiration and glycolytic rate, in splenocytes and bone marrow (BM) cells from R24-immunized or mock-immunized mice using Seahorse technology. The OCR of splenocytes and BM cells from R24-vaccinated mice was consistently higher than that measured in non-vaccinated mice (**Figure 4A**), indicating significantly increased ATP production, basal and maximal respiration, as well as spare respiratory capacity (**Figure 4B**). Proton Efflux Rates (PER) were also consistently higher in splenocytes and BM cells from R24 vaccinated mice in comparison to those from non-vaccinated mice (**Figure 4C**), indicating significantly elevated basal glycolysis in the BM and significantly higher compensatory glycolysis in both splenocytes and BM cells (**Figure 4D**). This suggested that immune cells in R24-vaccinated mice are in a state of hybrid metabolic state where cells can use both glycolysis and oxidative phosphorylation for their activity, as was previously described for BCG-induced trained immunity (Arts et al., 2016). As Ly6G^+^ neutrophils increased in spleens of R24-immunized mice (**Figure 2B**) and were important for their protection and cross-protection against systemic infections (**Figure 3E, F**), we next sorted neutrophils by FACS from either spleens or BMs of R24-vaccinated mice and performed a mitochondrial stress analysis using the Seahorse technology. Overall, we observed the greatest OCR changes induced by R24-mediated trained immunity in splenic, as opposed to BM, neutrophils (**Figure 4E**). In particular, both splenic and BM neutrophils increased their ATP production upon vaccination, while splenic but not BM neutrophils elevated their maximal respiration (**Figure 4F**). Finally, whereas R24-mediated training induced a significant elevation of spare respiratory capacity in splenic neutrophils, it caused a significant drop of the same capacity in neutrophils from the BM (**Figure 4F**).

Taken together, immunization *via* a live attenuated strain of *C. albicans*, obtained by serial passaging through the mouse gastrointestinal tract (Tso et al., 2018), induced numeric and metabolic changes in mouse neutrophils, which correlate with host protection against subsequent infection with a fully virulent, wild-type strain of *C. albicans*. In summary, neutrophils appear to play an important role in the trained immunity program elicited in mice by gut-evolved *C. albicans* strains.

## Discussion

Trained immunity is a type of innate immunological memory that has gained recent attention for its possible role in vaccination and as a potential therapeutic avenue to combat inflammatory and autoimmune diseases (Gyssens and Netea, 2019). Among the best-described PRR-mediated mechanisms of trained immunity are the Dectin-1 and the Nod2 pathway (Mulder et al., 2019). However, when we immunized mice devoid of either Dectin-1 or Nod2 with gut-evolved *C. albicans* R24, most of them survived subsequent infection with virulent *C. albicans* (**Figure 1**). There are two possible explanations for this observation: (i) either the gut-evolved *C. albicans* strain (R24) acted as a live attenuated vaccine that induced classical adaptive immunological memory against the virulent strain, or (ii) gut-evolved *C. albicans* strain induced a trained innate immunity mechanism that is independent of either Dectin-1 and/or Nod2. In our previous work we showed that immunization with gut-evolved *C. albicans* strains conferred protection against subsequent infections also in Rag1-KO mice, making it unlikely that classical adaptive immunity is of main importance in this system (Tso et al., 2018).

Monocytes, macrophages, NK cells and, to a lesser extent, innate lymphoid cells (ILCs) (Mulder et al., 2019) have been considered as the main cellular mediators of trained immunity. More recently, neutrophils have been described as potential mediators of trained immunity as well (Moorlag et al., 2020). The mass cytometry (CyTOF) data presented in this study indicate that Ly6G^+^ neutrophils doubled in frequency in the spleens of mice immunized with gut-evolved *C. albicans* compared to mice immunized with a sublethal dose of WT SC5314 *C. albicans* or mock-immunized mice (**Figure 2**). The CyTOF analysis occurred at a time point, 28 days post-immunization, when mice had fully recovered and fungal cells had cleared from circulation (Tso et al., 2018). Nevertheless, the difference in neutrophil cell numbers could potentially be attributable to differences in the doses used for immunization. Using a higher dose of the WT SC5314 strain to match the one used for the evolved strains would not be possible, as animals would succumb to the lethal infection within a few days. Our data thus highlights a potential role for trained neutrophils in driving R24-mediated host protection against secondary infections, albeit more studies would be required to fully demonstrate this.

A potential criticism to this work is that while the CyTOF analysis that revealed the numeric expansion of splenic neutrophils was performed at day 28 post-immunization, all mouse secondary challenge experiments were done at day 14. The reason for this choice is that in our previous work we showed that significant host protection against secondary challenge was observable already at earlier time points (Tso et al., 2018). However, the following evidence suggests that neutrophil numbers were expanded already at this earlier time point. First, we looked at the sorting data for the Seahorse experiments done on BM cells. At day 14 post-immunization with the R24 strain, the percentage of neutrophils among all live BM cells was 50.4 ± 3.8% (*N* = 3), while in mock-immunized mice it was 31.4 ± 0.6% (*N* = 2). While neutrophil numbers in the control group were in line with published reference values (between 19.81% to 34.67%, depending on mouse strain and gender (Hensel et al., 2019), the difference with R24-immunized mice was statistically significant (*P* < 0.05, two-tailed Student’s t-test). Moreover, in two independent sorting experiments, the relative frequency of neutrophils among all live splenocytes from R24-immunized mice was 10.1% and 10.9%, respectively, which is markedly higher than the published reference values (between 0.88% to 3.45%, depending on mouse strain and gender).

Using a combination of depletion strategies, we observed that phagocytes in general and neutrophils in particular were required for mice immunized with gut-evolved *C. albicans* to survive to subsequent infections by WT SC5314 *C. albicans*. Neutrophils play a crucial role in the primary defense against systemic infections, including candidemia (Fradin et al., 2005); hence, this observation could simply reflect this well-known fact. In order to prove that neutrophils are carriers of trained immunity, we would have to perform an adoptive transfer of neutrophils from immunized animals to test if this enables naïve hosts to survive an infectious challenge. Unfortunately, such experiments are inherently challenging due to the short half-life of neutrophils (Hidalgo et al., 2019). Moreover, clodronate liposomes deplete phagocytic cells in general, hence not only macrophages and neutrophils, but also monocytes (Sunderkotter et al., 2004). Monocytes mediate trained immunity by *C. albicans* and by the fungal cell wall component β-glucan (Quintin et al., 2012), hence they are very likely to play a role also in trained immunity by gut-evolved *C. albicans* strains. Interestingly, deletion of CCR2, which is important for mobilization of monocytes during inflammation (Tsou et al., 2007), only had a partial effect in reducing the protection of mice from lethal fungal challenge in R24-vaccinated mice. This confirms the important role that monocytes play in this system, but also suggest that other innate immune cells, e.g., neutrophils, might play a role here. It would be interesting, for instance, to investigate a possible interplay and cross-talk between monocytes and neutrophils in this system, which might explain the partial requirement of monocytes in this trained immunity model.

Trained immunity has been linked to metabolic reprogramming in immune cells (Cheng et al., 2014; Arts et al., 2016; Arts et al., 2016). Here we showed increased mitochondrial respiration and glycolysis in splenocytes and BM cells of R24 immunized mice, a “hybrid energetic” state that has been reported in BCG-trained monocytes (Arts et al., 2016). Of note, β-glucan trained monocytes and macrophages show a clear shift away from oxidative phosphorylation and towards aerobic glycolysis (Cheng et al., 2014). Altogether, these results suggest that different training stimuli induce different metabolic changes in different innate immune cells (**Table 1**). Understanding the functional consequences of these changes on the ability of innate immune cells to respond to pathogens will be of great importance to harness trained immunity for vaccination.

**Table 1:** Examples of different stimuli inducing different trained immunity pathways.

Training stimulus	Receptor required for training	Affected immune cell type(s)	Epigenetic changes	Metabolic changes	References
MCMV	Ly49H	NK cells	Increased chromatin accessibility at enhancer regions	Unknown	(Sun et al., 2009; Lau et al., 2018)
Sublethal doses of WT *C. albicans*	Unknown, possibly Dectin-1	Monocytes, macrophages	H3K4 trimethylation	Unknown	(Quintin et al., 2012)
β-glucan	Dectin-1	Monocytes, macrophages	H3K4 monomethylation, H3K27 acetylation	Shift from oxidative phosphorylation towards aerobic glycolysis	(Saeed et al., 2014; Cheng et al., 2014)
BCG	Nod2	NK cells, monocytes, neutrophils	H3K4 trimethylation	Increased mitochondrial respiration and glycolysis	(Kleinnijenhuis et al., 2012; Kleinnijenhuis et al., 2014; Arts et al., 2016; Moorlag et al., 2020)
Gut-evolved *C. albicans*	Unknown	Neutrophils, and to a lesser extent, monocytes	Unknown	Increased mitochondrial respiration and glycolysis	This study

Moreover, besides increased neutrophil numbers in the spleen (**Figure 2B**), we also observed increased respiratory capacity in splenic neutrophils 14 days after immunization with a gut-evolved *C. albicans* strain (**Figures 4E, F**). How could such a cellular phenotype persist so long after the primary infection, given the short lifespan of neutrophils? The answer might lie in the bone marrow. Indeed, others have reported long-lasting BCG-induced effects in BM-resident hematopoietic stem cells (Kaufmann et al., 2018). Consistent with our own observation of metabolic changes in the BM of R24-trained mice (**Figures 4A–D**), we could thus speculate that metabolic changes in BM-resident long-lived neutrophil precursors might be ‘inherited’ by short-lived neutrophils developing from such precursors.

The final question is whether these metabolic changes are truly important to confer a trained immunity phenotype to the neutrophils and how these trained neutrophils mediate protection against host infections. Although further studies would be required to fully answer these questions, we could make some speculations based on recent publications. Moorlag *et al.* reported that BCG vaccination induces long-term phenotypic changes in human neutrophils, including increased expression of activation markers, augmented secretion of pro-inflammatory cytokines and enhanced antimicrobial activity against unrelated pathogens (Moorlag et al., 2020). Mouse neutrophils trained by gut-evolved *C. albicans* might therefore protect the host either directly *via* enhanced phagocytic or antimicrobial activity or indirectly *via* recruitment of other immune cells. Functional changes reported by Moorlag et al. in trained human neutrophils were associated with epigenetic changes, such as modifications in histone methylation (Moorlag et al., 2020). Prior work has already established that histone methylation changes in BCG-vaccine driven trained human monocytes were in fact dependent on metabolic changes in glycolytic pathways (Arts et al., 2016). Crosstalk and interdependence between metabolic and epigenetic reprogramming might in fact be a more general feature of trained immunity, as it was observed also in monocytes exposed to β-glucan (Arts et al., 2016). It is thus tempting to hypothesize that the metabolic changes in mouse neutrophils described in current paper might be linked to the epigenetic changes observed before (Moorlag et al., 2020).

In conclusion, this study highlights that at least some training stimuli (here: gut-evolved *C. albicans*) induce both numeric and metabolic changes in neutrophils. While we require further research to test the extent to which these changes are important for trained immunity and for protecting hosts from secondary infections, these results shed light on the broad effects that training has on the innate immune system as a whole. The similarities and differences in metabolic changes induced by different stimuli in different cell types highlighted in this and other studies (**Table 1**) suggest the existence of more than one mechanism of trained immunity.

## Data Availability Statement

The original contributions presented in the study are included in the article/**Supplementary Material**. Further inquiries can be directed to the corresponding authors.

## Ethics Statement

All procedures involving animals were approved by the Institutional Animal Care and Use Committee (IACUC) of A*STAR (Biological Resource Centre (BRC), Biopolis, Singapore) in accordance with the guidelines of the Agri-Food & Veterinary Authority (AVA) and the National Advisory Committee for Laboratory Animal Research (NACLAR).

## Author Contributions

JR-C, GT, and NP designed the study. JR-C, GT, AT, PH, and JB performed the experiments. KT and EN performed and analyzed the CyTOF experiments. JR-C and NP analyzed the data and wrote the paper. JR-C, NP, AS, and EN edited and reviewed the final manuscript, in accordance with all authors. All authors contributed to the article and approved the submitted version.

## Funding

This study was supported by Agency for Science, Technology, and Research (A*STAR) Investigatorship awards JCO/1437a00117 to NP, JCO/#15302FG151 to AS; Immunomonitoring platform grant (#BMRC/IAF/311006, #H16/99/b0/011, and #NRF2017_SISFP09); and by core funding from SIgN.

## Conflict of Interest

EN is a co-founder, advisor and shareholder of ImmunoScape Pte. Ltd. EN is an advisor for Neogene Therapeutics and Nanostring Technologies. NP is employed by F. Hoffmann-La Roche AG.

The remaining authors declare that the research was conducted in the absence of any commercial or financial relationships that could be construed as a potential conflict of interest.

## Publisher’s Note

All claims expressed in this article are solely those of the authors and do not necessarily represent those of their affiliated organizations, or those of the publisher, the editors and the reviewers. Any product that may be evaluated in this article, or claim that may be made by its manufacturer, is not guaranteed or endorsed by the publisher.

## Supplementary Material

Supplementary Figure 1 Immunization of Dectin1-KO mice against systemic candidiasis using the gut-evolved *C. albicans* strain R24. Ten Dectin1-KO mice were immunized with the live attenuated *C. albicans* strain R24 and their survival was monitored for 28 days.

Supplementary Figure 2 Cytometry by Time-Of-Flight (CyTOF) analysis of splenocytes from mice vaccinated with the evolved strains R24 and W2N. **(A–E)** Bar graphs show the average frequency of the different populations in the splenocytes from vaccinated and non-vaccinated mice. No significant changes were detected between groups using One-way ANOVA with multiple comparisons.

Supplementary Figure 3 Depletion of different immune populations of mice immunized with gut-evolved *C. albicans* strain R24. **(A)** % of CD11b^+^/Cd11c^+^ and Ly6c+ cells after depletion with Clodronate liposomes **(B)** % of Ly6c^+^/F4/80^+^ cells after depletion with CSF1R neutralizing antibody **(C)** % of NK1.1^+^ cells after depletion with NK1.1 neutralizing antibody **(D)** % of Ly6C^high^/Cd11b^+^ cells after depletion with Ly6G neutralizing antibody.
