# Supplementary figures and images for: Gut-Evolved Candida albicans Induces Metabolic Changes in Neutrophils
Source: Front Cell Infect Microbiol. 2021 Nov 22;11:743735. doi: 10.3389/fcimb.2021.743735 (PMC8645939; doi:10.3389/fcimb.2021.743735)

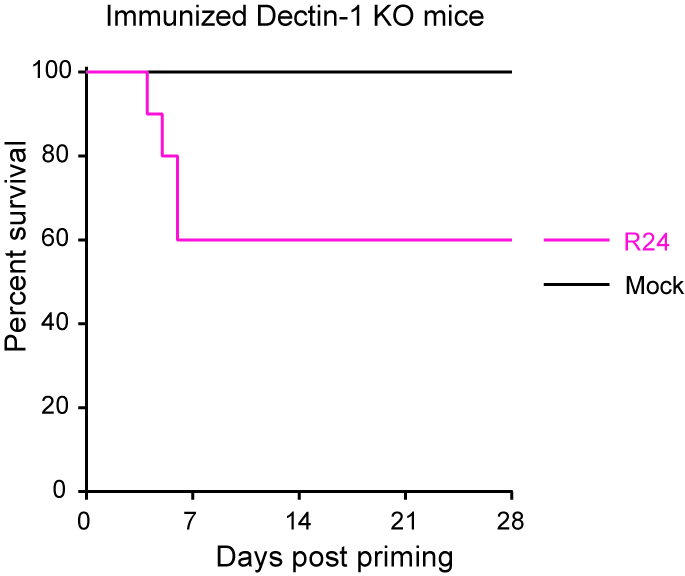

Supplement: Supplementary Figure 1 — Immunization of Dectin1-KO mice against systemic candidiasis using the gut-evolved C. albicans strain R24. Ten Dectin1-KO mice were immunized with the live attenuated C. albicans strain R24 and their survival was monitored for 28 days. [file Image_1.tif]

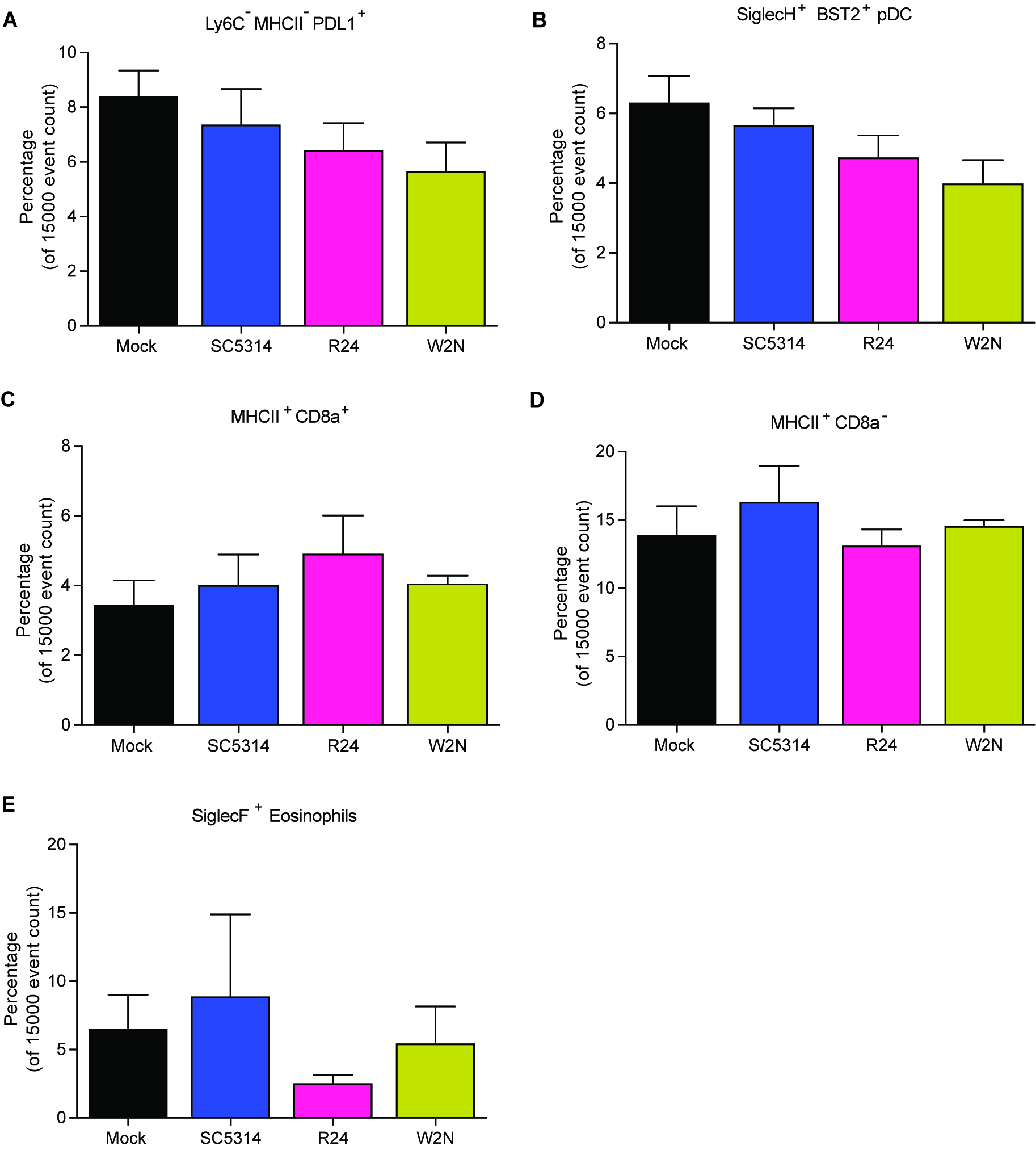

Supplement: Supplementary Figure 2 — Cytometry by Time-Of-Flight (CyTOF) analysis of splenocytes from mice vaccinated with the evolved strains R24 and W2N. (A–E) Bar graphs show the average frequency of the different populations in the splenocytes from vaccinated and non-vaccinated mice. No significant changes were detected between groups using One-way ANOVA with multiple comparisons. [file Image_2.tif]

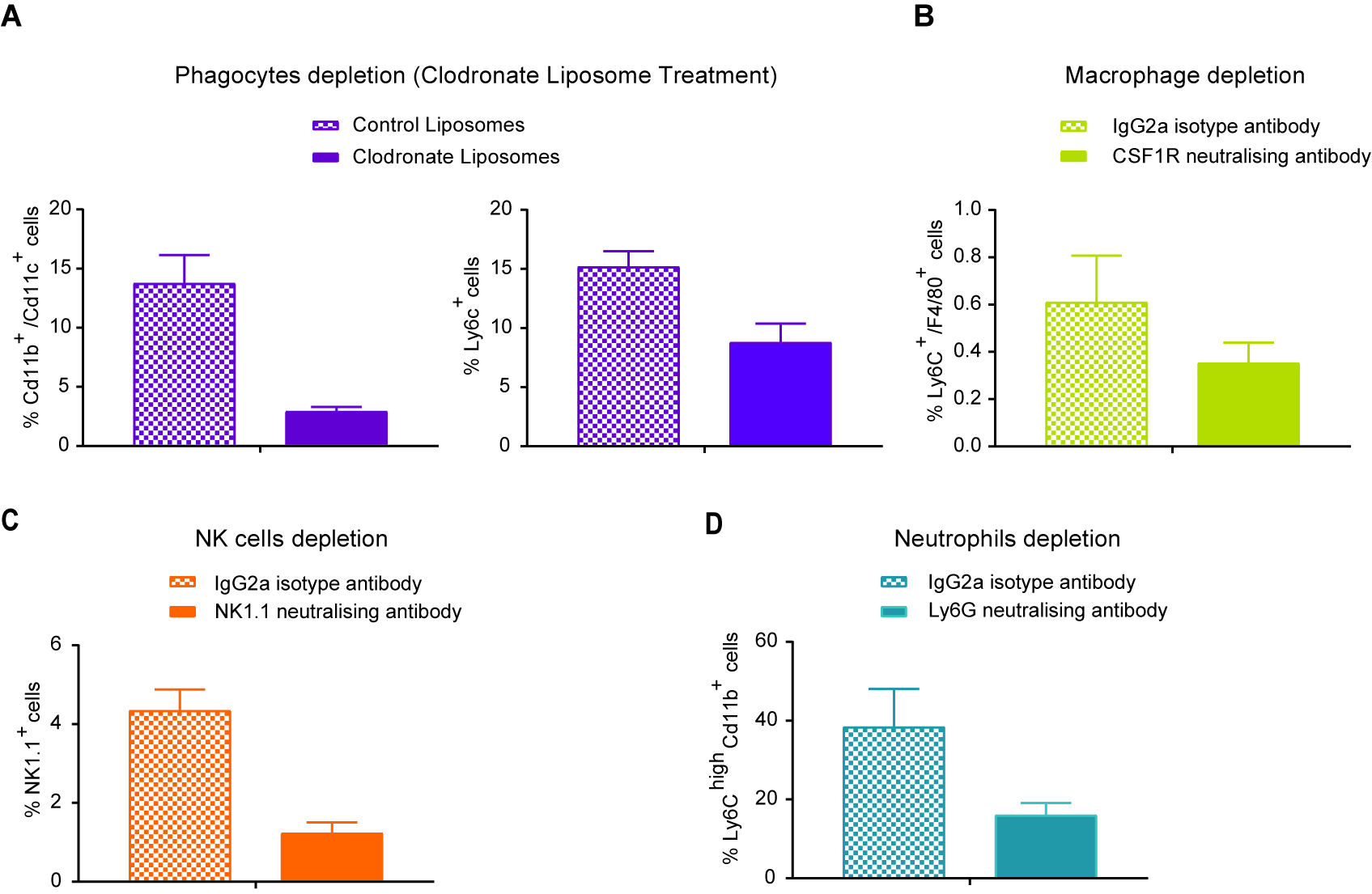

Supplement: Supplementary Figure 3 — Depletion of different immune populations of mice immunized with gut-evolved C. albicans strain R24. (A) % of CD11b+/Cd11c+ and Ly6c+ cells after depletion with Clodronate liposomes (B) % of Ly6c+/F4/80+ cells after depletion with CSF1R neutralizing antibody (C) % of NK1.1+ cells after depletion with NK1.1 neutralizing antibody (D) % of Ly6Chigh/Cd11b+ cells after depletion with Ly6G neutralizing antibody. [file Image_3.tif]
