# Supplementary material for: Gut-Evolved Candida albicans Induces Metabolic Changes in Neutrophils
Source: Front Cell Infect Microbiol. 2021 Nov 22;11:743735. doi: 10.3389/fcimb.2021.743735 (PMC8645939; doi:10.3389/fcimb.2021.743735)
Supplement: Supplementary file 4 [file Table_1.docx]

Table S1. List of antibodies used for CyTOF

| **Metal** | **Antibody** | **Clone** | **Cat number** | **Company** |
| --- | --- | --- | --- | --- |
| 89 | CD45 | 30-F11 | 3089005B | Fluidigm |
| 112/114 | CD19 | 6D5 | Q10379 | Invitrogen |
| 141 | CD43 | S7 | 553268 | BD Biosciences |
| 142 | MHCII | Y-3P | BE0178 | BioXCell |
| 143 | B220 | RA3-3A1/6.1 | BE0067 | BioXCell |
| 144 | CD11a | FD441.8 | BE0005-1 | BioXCell |
| 145 | Ly6GC | RB6-8C5 | 553122 | BD Biosciences |
| 146 | CD8 | 53-6.7 | 558733 | BD Biosciences |
| 147 | Ly6G | 1A8 | 551459 | BD Biosciences |
| 148 | Ly6c | HK1.4 | 128002 | Biolegend |
| 149 | CD4 | H129.19 | 553647 | BD Biosciences |
| 150 | CX3CR1 | SA011F11 | 149002 | Biolegend |
| 151 | CD62L | MEL-14 | 104402 | Biolegend |
| 152 | CD11c | N418 | 117302 | Biolegend |
| 153 | CD11b | M1/70 | 557394 | BD Biosciences |
| 154 | CD3 | 145-2C11 | 100302 | Biolegend |
| 155* | CD86 | GL1 | 553689 | BD Biosciences |
| 156 | BST2 | PDCA-1 |  | Purified in house |
| 157* | MerTK Biotin (Primary) | Accession number: 60805 | BAF591 | R&D Systems |
|  | Streptavidin (Secondary) |  |  | Synthesized in house |
| 158 | TER119 | TER-119 | 116202 | Biolegend |
| 159 | F4/80 | C1:A3-1 | MCA497GA | Bio-Rad |
| 160 | FcER1 | MAR-1 | 14-5898-82 | eBioscience |
| 161* | CD25 | PC61 | 102002 | Biolegend |
| 162* | PD-L1 | 10F.9G2 | 124302 | Biolegend |
| 163* | PD-L2 | TY25 | 107202 | Biolegend |
| 164 | CD103 | 2E7 | 14-1031-82 | Biolegend |
| 165 | CD64 | X54-5/7.1 | 108402 | Biolegend |
| 166 | CD48 | HM48-1 | 103402 | Biolegend |
| 167 | CD49b | DX5 | 108902 | Biolegend |
| 168 | CD44 | IM7 | 103002 | Biolegend |
| 169 | CCR2 APC (Primary) | 475301 | FAB5538A | R&D Systems |
|  | anti-APC (Secondary) | APC003 | 408002 | Biolegend |
| 170 | CD38 | 90 | 102723 | Biolegend |
| 171 | Siglec H | 551 | 129602 | Biolegend |
| 172 | Sca-1 | D7 | 557403 | BD Biosciences |
| 173* | CD172 | P84 | 552371 | BD Biosciences |
| 174 | CD24 | M1/69 | 101802 | Biolegend |
| 175 | Siglec F | E50-2440 | 552125 | BD Biosciences |
| 176 | CD90 | T24/31 | BE0212 | BioXCell |
